# Isotope-based water-use efficiency of major greening plants in a sponge city in northern China

**DOI:** 10.1371/journal.pone.0220083

**Published:** 2019-07-25

**Authors:** Changchao Li, Miansong Huang, Jian Liu, Shuping Ji, Ruiqi Zhao, Di Zhao, Ruilian Sun

**Affiliations:** 1 Institute of Environmental Research, Shandong University, Qingdao, China; 2 Ningxia Capital Sponge City Construction & Development Co., Ltd, Guyuan, China; 3 Beijing Capital Co., Ltd, Beijing, China; Qingdao Agricultural University, CHINA

## Abstract

To tackle urban water issues, the Chinese government has promoted the construction of sponge cities in recent years. Thirty cities have been designated as experimental sites to serve as models for future sponge city construction, as more than 80% of the built-up urban areas in China must reach the standards of sponge cities by 2030. Greening plants play an important role in sponge cities, and water-use efficiency (WUE) is a vital index to determine whether plants could adapt to and grow healthily in environments with water deficits. In this study, WUE of greening plants was quantified by measuring the stable carbon isotope fractionation. Suitable plants for the green spaces in Guyuan sponge city, in northern China, were selected based on their WUE, and the main factors affecting WUE were studied in four habitats within the city. Plant species identity had the greatest effect on WUE, while habitat and plant life form had lower effect, illustrating that WUE is a relatively stable and reliable index for the classification of plant species. We can improve the WUE and ecological function of green spaces in sponge cities by using isotope technology to select suitable plant species with high WUE. To our knowledge, this study is the first to select plant species for sponge city by using this method, providing a quick and scientific method for the selection of greening plants for future sponge cities.

## Introduction

Sponge city is a new type of urban stormwater management concept. It is based on the premise that, like sponges, cities should be “elastic” in order to adapt to environmental changes and cope with natural disasters [1]. During rainfall, the sponge city absorbs, accumulates, seeps, and purifies water. When needed, it releases and uses the stored water [1]. The concept of sponge cities in China is similar in function to Low Impact Development, Sustainable Drainage Systems, and Best Management Practice in other countries. However, this concept draws on internationally successful construction experience and practice cases, while taking into account the local conditions, climate, geography, and other factors specific to China, so it has more comprehensive connotations and far-reaching significance [2]. In April 2015, China designated 16 cities as the first batch of experimental sites for sponge cities; in April 2016, 14 additional cities were designated as the second batch of sponge city experimental sites. The construction of sponge cities will promote the transformation and development of China’s traditional stormwater management and serves as the benchmark and guidelines for future urban rainwater constructions in China [3].

Greening plants form an important part of urban ecosystems. In sponge cities, greening plants can absorb and utilize nitrogen, phosphorus, and potassium (present in precipitation and residential drainage) as nutrients; they can absorb and degrade pesticides and metal ions, and purify water by increasing the presence of dissolved oxygen [4]. Plants can reduce runoff and peak flow by intercepting precipitation [5], increasing rainwater seepage and evaporation, and increasing groundwater recharge. The greening plants can also absorb dust particles, reduce noise, and beautify the environment, thus, giving residents a more comfortable living environment [6, 7].

Water-use efficiency (WUE) is an important index of sponge city greening plants, especially for sponge cities in arid regions. Plants with high WUE have strong drought tolerance, which can reduce the need for artificial irrigation, lower maintenance costs, and increase urban ecosystem services [8, 9]. At present, the methods for determining the WUE of plants include field measurement, leaf gas exchange technology, and stable carbon isotope technology [10, 11]. The stable carbon isotope ratio (denoted as δ^13^C) is positively correlated with the WUE of a plant [12, 13], and larger δ^13^C values imply higher WUE [14–16]. Therefore, δ^13^C can be used as an evaluation index for plant WUE [17, 18]. The advantages of stable carbon isotope technology are that (1) it allows for long-term measurements, overcoming the shortcomings of conventional methods that can only study short-term and instantaneous WUE, (2) it limits the destruction of plants during sampling, (3) it is not limited by factors such as sampling time, sampling location, and the underlying surface conditions of study area, and (4) the plant material is convenient for storage and measurement [19]. Since stable carbon isotope technology has become a recognized method for estimating the long-term WUE of plants [20], we used this method to measure the WUE of greening plants in the study area.

Studies on the WUE of plants by using stable carbon isotope technology have shown a strong correlation between the δ^13^C value and WUE with minimal interference due to other factors [21, 22]. Carbon isotope technology has been used to estimate the changes in the WUE of grasslands in the past decades [23, 24]. Different plant genotypes have different responses to water availability [25]; therefore, the δ^13^C value is an effective index for selecting genotypes with higher WUE [26]. There are several studies on the selection or evaluation of greening plants in sponge cities [27–29]. For example, waterlogging resistance and drought-tolerant of greening plants in Shenzhen sponge city have been studied to provide guidelines for the construction of other sponge cities [27, 28]. The growth adaptability and flood tolerance of six common herbaceous garden plants have been compared to provide a screening method for the selection of greening plants in sponge cities [29]. However, to our knowledge, there is no study on the WUE of greening plants in sponge cities based on isotope technology.

The city of Guyuan is in the transition zone between the semi-arid to the arid and moderate temperate zone of China. Because of global climate change, the ecological environment in Guyuan has become more fragile in recent years [30]. As a result, natural disasters are more frequent, and drought is the most prominent environmental phenomenon [30]. Moreover, water deficit has become the dominant factor restricting agricultural production in Guyuan. In 2016, the city of Guyuan was selected to form a part of the second batch of sponge city experimental sites in China, aiming to solve the water shortage through the construction of a sponge city. Sponge cities are constructed mainly to solve two types of urban ecological problems, waterlogging and water shortage, and Guyuan is a representative of cities with water shortages. Therefore, in Guyuan, greening plants with high WUE should be identified and chosen based on their ability to adapt to the environment and boost the ecological functions of the sponge city.

The objectives of this study were (1) to explore the main factors affecting WUE of greening plants in Guyuan, (2) to explain whether the WUE of green spaces in sponge cities can be improved by utilizing plant species with high WUE, and (3) to provide a convenient and scientific method for selecting greening plants for sponge cities.

## Materials and methods

### Ethics statement

We collected soil and plant samples for our study with the official permission of Ningxia Capital Sponge City Construction & Development CO., LTD. There is no endangered or protected plant species were sampled.

### Study area

The study area is in Guyuan, a city of roughly 1.23 million people and approximately 10540 km^2^ in area. The city is located in the south of the Ningxia Hui Autonomous Region (35° 14′ N-36° 32′ N, 105° 19′ E-106° 58′ E). The southern part of Guyuan is a semi-humid zone in the middle temperate zone.

The annual average temperature in Guyuan is 6.9°C and the annual average precipitation is low (166.9–647.3 mm) [31]. The precipitation of Guyuan is more in the south and less in the north and the distribution between seasons is uneven, with heavy rainfall in summer and autumn, and less rainfall in winter and spring [31]. Guyuan is situated at a high altitude, has good atmospheric transparency, high radiation intensity, long sunshine hours, and abundant illumination resources [32].

### Sample collection and preparation

**Fig 1:**
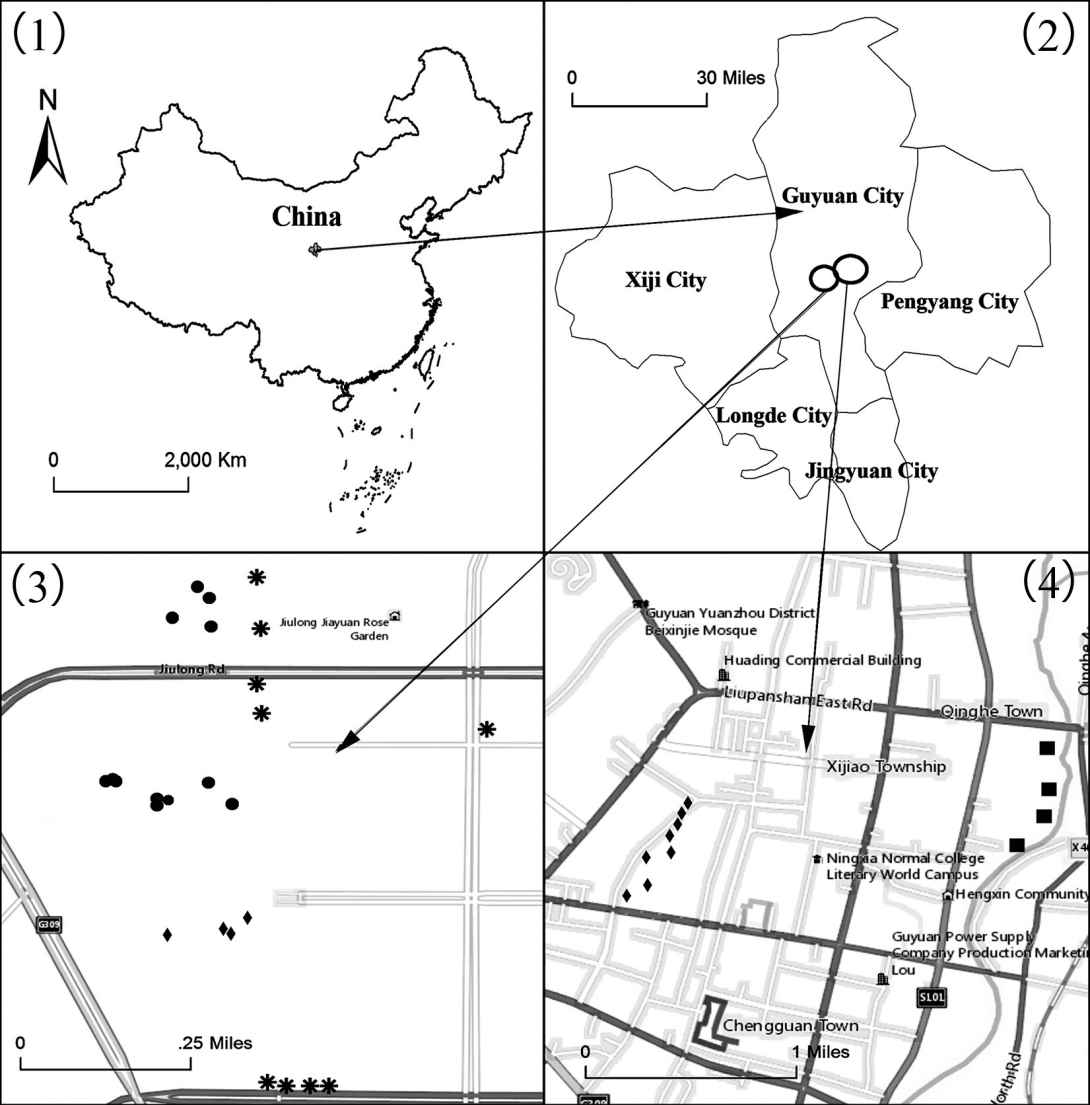
Map of sampling plots collected from Guyuan. (1) The map of China; (2) the map of Guyuan City; and the locations of the sampling plots are shown in (3) and (4). The circles: the sampling plots of residential green spaces; the asterisks: the sampling plots of street green spaces; the diamond: the sampling plots of park green spaces; the squares: the sampling plots of the riverside wetland.

Four main habitats of greening plants were chosen, namely, residential green spaces (Denoted by A), street green spaces (Denoted by B), park green spaces (Denoted by C), and the riverside wetland (Denoted by D) in which twelve, nine, twelve, and four sampling plots were selected, respectively, as shown in Fig 1, and the geographic coordinates of all the 37 sampling plots were listed in S1 Table.

In each plot, mixed topsoil (0–20 cm) was collected from five subsamples. Half of the topsoil from each subsample was stored in an ice bucket for determination of ammonium nitrogen (NH_4_^+^-N) content and nitrate nitrogen (NO_3_^−^-N) content in the soil, and the other half was stored at room temperature for determination of other soil indexes, including soil pH, soil organic matter (SOM), total nitrogen (TN), and total phosphorus (TP) content.

In each plot, depending on leaf size, 5 to 20 healthy and complete leaves (mostly 10) of each plant species were picked and placed into a coded envelope until further analysis. A total of 32 plant species were collected in 37 plots, with at least four replicates per species. The 32 plant species were numbered from 1 to 32, and the species names corresponding to each species number were listed in S2 Table.

### Measurement

The soil NH_4_^+^-N content and NO_3_^−^-N content were extracted with a 2 mol·L^−1^ KCl solution at 1:5 (soil:water) and measured with a continuous-flow auto-analyzer (AA3, Seal Analytical Inc., Southampton, UK). Soil pH was measured in a 1:2.5 (soil:water) mixture using a glass electrode pH meter (PHS-2F, Shanghai INESA Instrument Co., Ltd, China). The SOM was determined with the K_2_Cr_2_O_7_-H_2_SO_4_ wet oxidation method, the soil TN content was determined with the Kjeldahl method using an automatic Kjeldahl analyzer (Haineng-K9860, Hanon Instruments Co., Ltd., Jinan, China), and the soil TP content was determined using acid-soluble molybdenum antimony blue colorimetry with an ultraviolet-visible spectrophotometer (UV-5500, Shanghai Metash Instruments Co., Ltd., China) [33].

The leaves of the plants were rinsed with deionized water, blotted with tissue paper to remove the water completely, then treated at 115°C for 15 minutes for deactivation of enzymes, and oven dried at 70°C for 48 hours. The dried leaf samples were ground, sieved, and measured with a stable isotope ratio mass spectrometer (IsoPrime100; Elementar, Germany) to obtain the δ^13^C values of plants surveyed.

### Quantifying WUE from δ^13^C

Farquhar [33] derived the relationship between WUE and δ^13^C value as follows:

$$WUE=\frac{\left(1-\theta )C_{a}(b-\delta_{a}+\delta_{p}\right)}{(b-a)1.6VPD},$$ (1)

where *a* is the fractionation occurring due to diffusion in air (4.4 ‰), *b* is the net fractionation caused by carboxylation (27 ‰) [23, 34]; *C*_*a*_ is the concentration of atmospheric CO_2_ (390 μL·L^−1^) [35]; *δ*_*a*_ is the δ^13^C value of air (-8.8 ‰) [36], *δ*_*p*_ is the δ^13^C value of the plant; *θ* is the ratio of carbon consumed by respiration in leaves and other organs throughout the growing season (0.3) [37]; and 1.6 is the ratio of diffusivities of water vapor and CO_2_ in air [24, 38]; and *VPD* is the vapor pressure deficit between the inside and outside of the leaf, that was calculated from the daily average meteorological data on the sampling date [39, 40]:

$$VPD=0.611\times 10^{17.502T/(240.97+T)}\times (1-RH),$$ (2)

where *T* is the temperature of leaf,°C, set *T* to the average temperature of the leaves when sampling (20°C); *RH* is atmospheric relative humidity, set it to the average relative humidity when sampling; 0.611 is saturated vapor pressure on a pure horizontal surface at 0°C. Substituting (2) into (1),

$$WUE=\frac{\left(1-\theta )C_{a}(b-\delta_{a}+\delta_{p}\right)}{\left(b-a)\times 1.6\times 0.611\times 10^{\frac{17.502T}{240.97+T}}\times (1-RH\right)},$$ (3)

*Ca* = 390 μL·L^−1^; *θ* = 0.3; *a* = 4.4 ‰; *b* = 27 ‰; *δ*_*a*_ = -8.8 ‰; *δ*_*p*_ = δ^13^C; *T* = 20°C; *RH* = 0.6.

Substituting all the data will yield the relationship between WUE and δ^13^C values:

$$WUE=50.12+1.4\;\delta^{13}C.$$ (4)

### Data analysis

Differences in soil indexes were analyzed using a one-way ANOVA with a post hoc Duncan test. The interaction effect of habitats and species identity on WUE were analyzed using two-way ANOVA. Differences in WUE between trees, shrubs, and herbs, and between evergreen and deciduous plants were analyzed using nonparametric tests. The tests were performed using IBM SPSS Statistics 22. A probability of 0.05 or lower was considered as significant in testing the null hypothesis of no differences in WUE and in other calculated values. Intraspecific differences in plants were calculated by the coefficient of variation using Microsoft Excel 2016. The map of sampling plots was drawn using ArcGIS 10.2.2, and other graphs were drawn using Origin 2018.

## Results

### Soil condition

The results of soil pH, SOM content, TP content, TN content, NH_4_^+^-N content, and NO_3_^−^-N content were list in Table 1, and the soil samples were evaluated according to the results of the second national soil survey in China. Thus, soil fertility was divided into six levels, with the first level being the most fertile and the sixth level the most barren. Accordingly, the soil pH values in Guyuan are comparatively higher. The SOM was generally low, except for the soil samples in habitat D that reached the fourth-level soil standard, and the SOM in the other three habitats is lower than the fourth-level soil standard. The TN content of all the soils was less than 0.5 g·kg^−1^, so all the soils belong to the sixth-level soil standard. The soil TP content was at the fourth-level soil standard. The NH_4_^+^-N and NO_3_^−^-N content was also generally low. Thus, the soil in the habitats of the main greening plants was alkaline and barren.

**Table 1:** Soil indexes in study area.

Habitat	pH	SOM	TP	TN	NH_4_^+^-N	NO_3_^−^-N
		(g·kg^−1^)	(g·kg^−1^)	(g·kg^−1^)	(mg·kg^−1^)	(mg·kg^−1^)
A	8.69±0.14a	7.52±2.23b	0.54±0.05a	0.26±0.06b	5.39±1.08a	1.33±0.56a
B	8.68±0.10a	6.65±1.06b	0.53±0.02a	0.25±0.04b	4.72±0.28a	1.27±0.99a
C	8.73±0.07a	6.11±1.47b	0.59±0.08a	0.24±0.03b	9.09±14.04a	6.00±14.00a
D	8.34±0.19b	10.37±5.62a	0.56±0.09a	0.35±0.16a	9.25±3.78a	0.95±0.16a

Values are expressed as mean ± standard deviations. A, B, C and D represent residential green spaces, street green spaces, park green spaces and the riverside wetland respectively. SOM: Soil organic matter; TP: Total phosphorus; TN: Total nitrogen; NH_4_^+^-N: Ammonium nitrogen; NO_3_^−^-N: Nitrate nitrogen; Different labels (a and b) are significantly different between four habitats (P < 0.05).

### Effects of habitats on WUE

**Fig 2:**
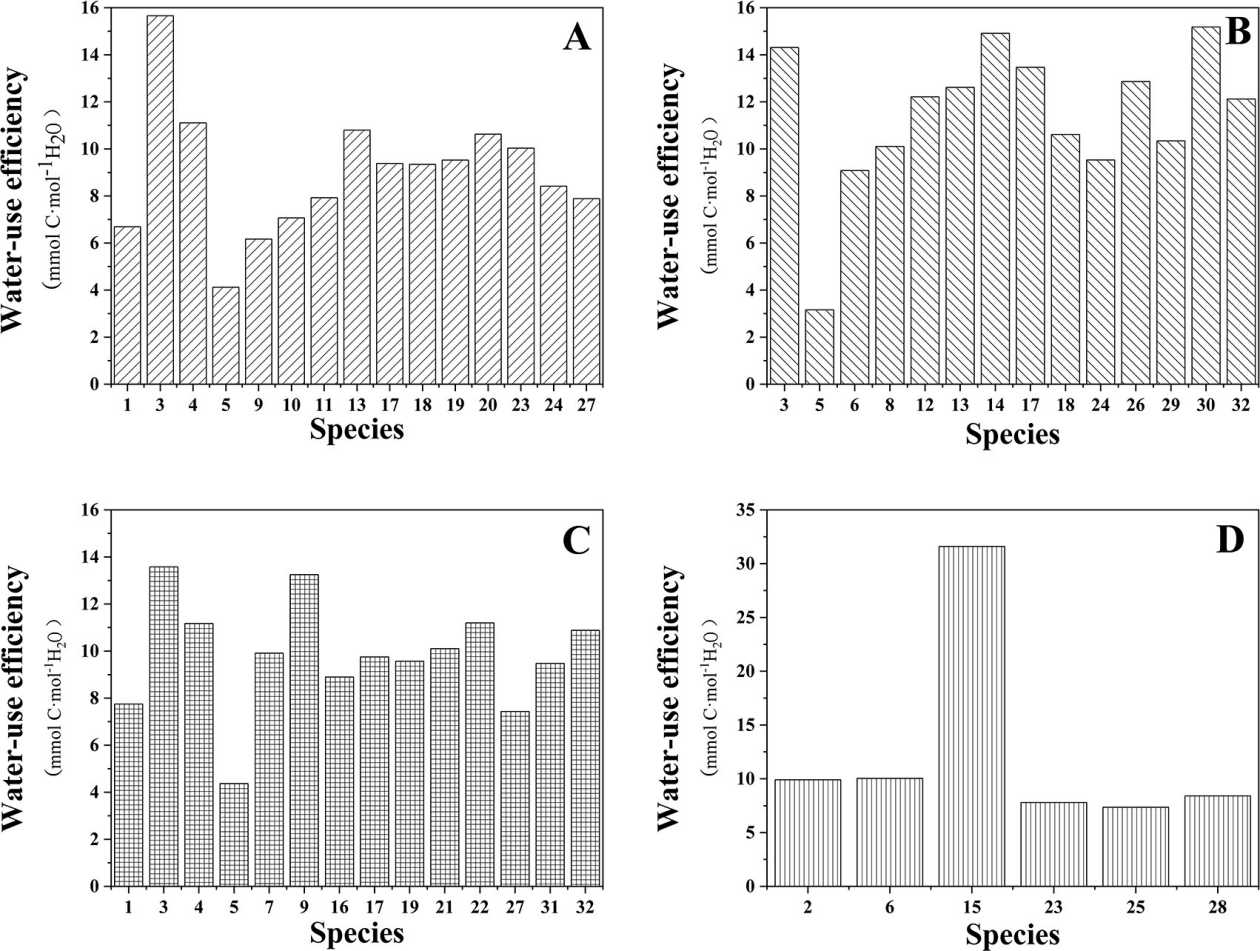
The WUE of the plant species surveyed in each habitat. A, B, C and D represent residential green spaces, street green spaces, park green spaces and the riverside wetland respectively. Plant species are represented by numbers on abscissa axis: 1-*Verbena officinalis* L., 2-*Cirsium setosum* (Willd.) Besser ex M.Bieb., 3-*Picea asperata* Mast., 4-*Dianthus chinensis* L., 5-*Ulmus pumila* ‘Jinye’, 6-*Rumex acetosa* L., 7-*Rosa chinensis* Jacq. var. *spontanea* (Rehd. et Wils.) Yü et Ku, 8-*Tamarix ramosissima* Lbd., 9-*Iris tectorum* Maxim., 10-*Prunus × cisterna* ‘Pissardii’, 11-*Zoysia tenuifolia*, 12-*Sedum spectabile*, 13-*Amygdalus triloba* (Lindl.) Ricker, 14-*Juniperus formosana* Hayata, 15-*E*. *crus-galli*, 16-*Lavandula angustifolia* Mill., 17-*Prunus Cerasifera Ehrh*. *f*. *atropurpurea* (Jacq.) Rehd., 18-*Trifolium repens* L., 19-*Iris lacteal Pall*. *var*. *chinensis* (Fisch.) Koidz., 20-*Rosa xanthina* Lindl., 21-*Euonymus phellomanus* Loes., 22-*Hosta ventricosa* (Salisb.) Stearn, 23-*Salix matsudana* Koidz., 24-*Lythrum salicaria* L., 25-*Typha orientalis* C. Presl, 26-*Platycladus orientalis* (L.) Franco, 27-*Rudbeckia hirta* L., 28-*Artemisia mongolica* (Fisch. ex Besser) Fisch. ex Nakai, 29-*Salix babylonica* L., 30-*Forsythia viridissima* Lindl., 31-*Coreopsis lanceolata* L., 32-*Syringa oblata* Lindl.

**Fig 3:**
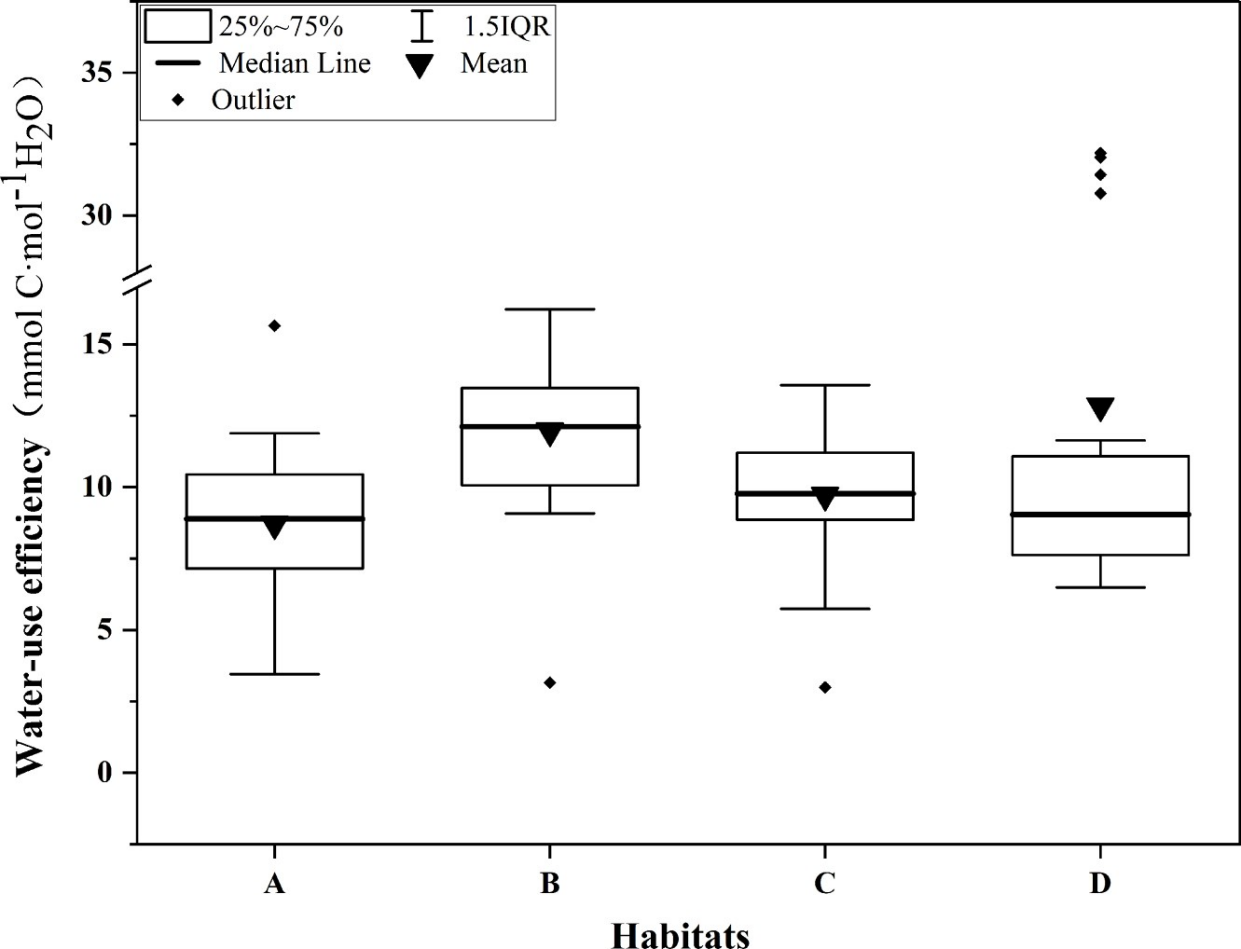
The WUE conditions of the four habitats. A, B, C and D represent residential green spaces, street green spaces, park green spaces and the riverside wetland respectively.

**Fig 4:**
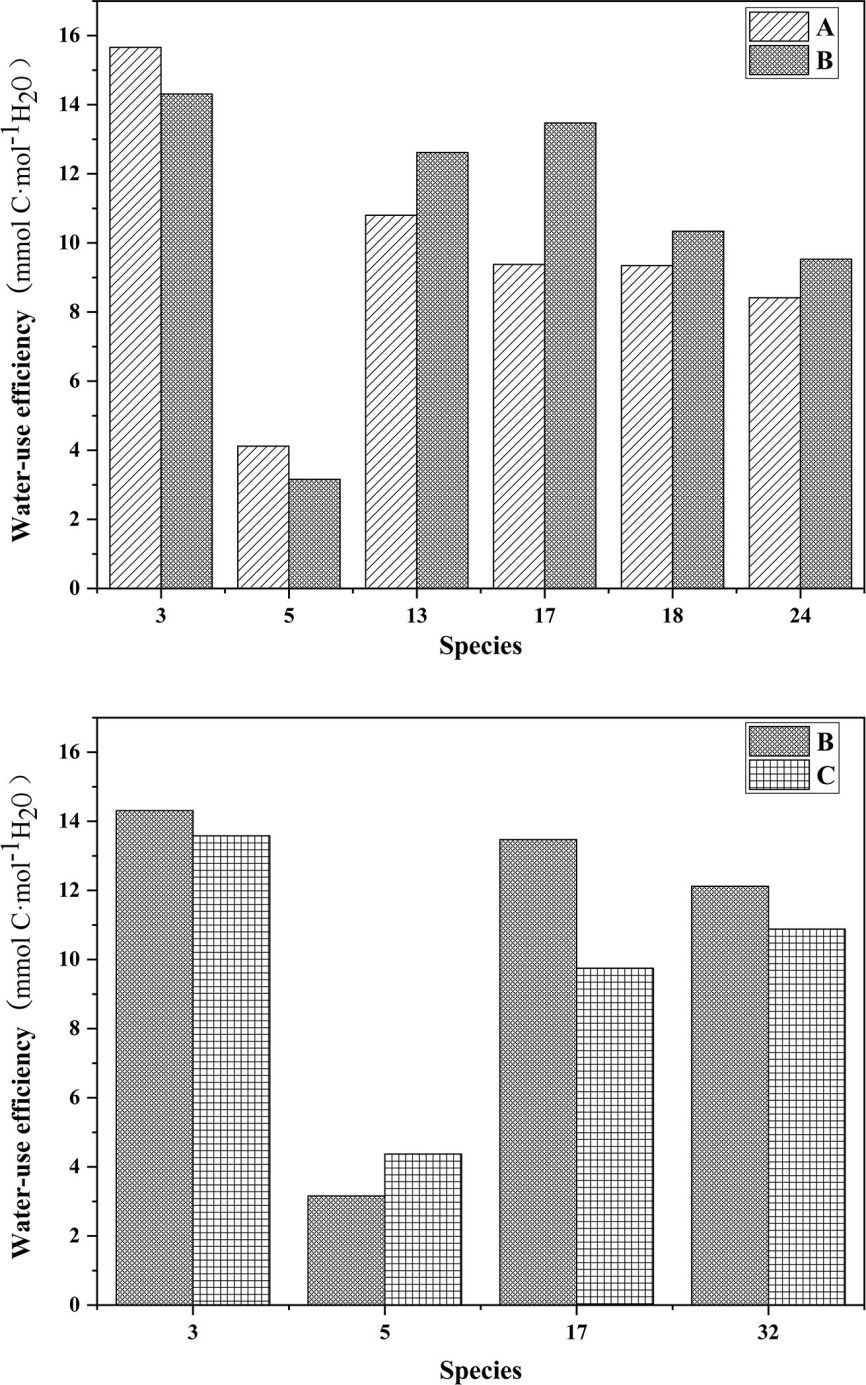
**The WUE of the same species in A—B and in B—C.** A, B, and C represent residential green spaces, street green spaces and park green spaces respectively. The names of the species corresponding to the species numbers are shown in the notes of Fig 2.

The WUE values of different plant species in each habitat are shown in Fig 2, and the WUE conditions of the green spaces in the four habitats are shown in Fig 3. The results of one-way ANOVA showed that there were significant differences in the WUE of different habitats (P < 0.001); the WUE values of habitats B and D were significantly higher than those of habitat A and C (P < 0.05). The WUE of the same plant species in habitat A and B and in habitat B and C were found out and shown in Fig 4. It was found that the WUE of same plant species between different habitats did not differ significantly.

### Effects of species identity on WUE

**Fig 5:**
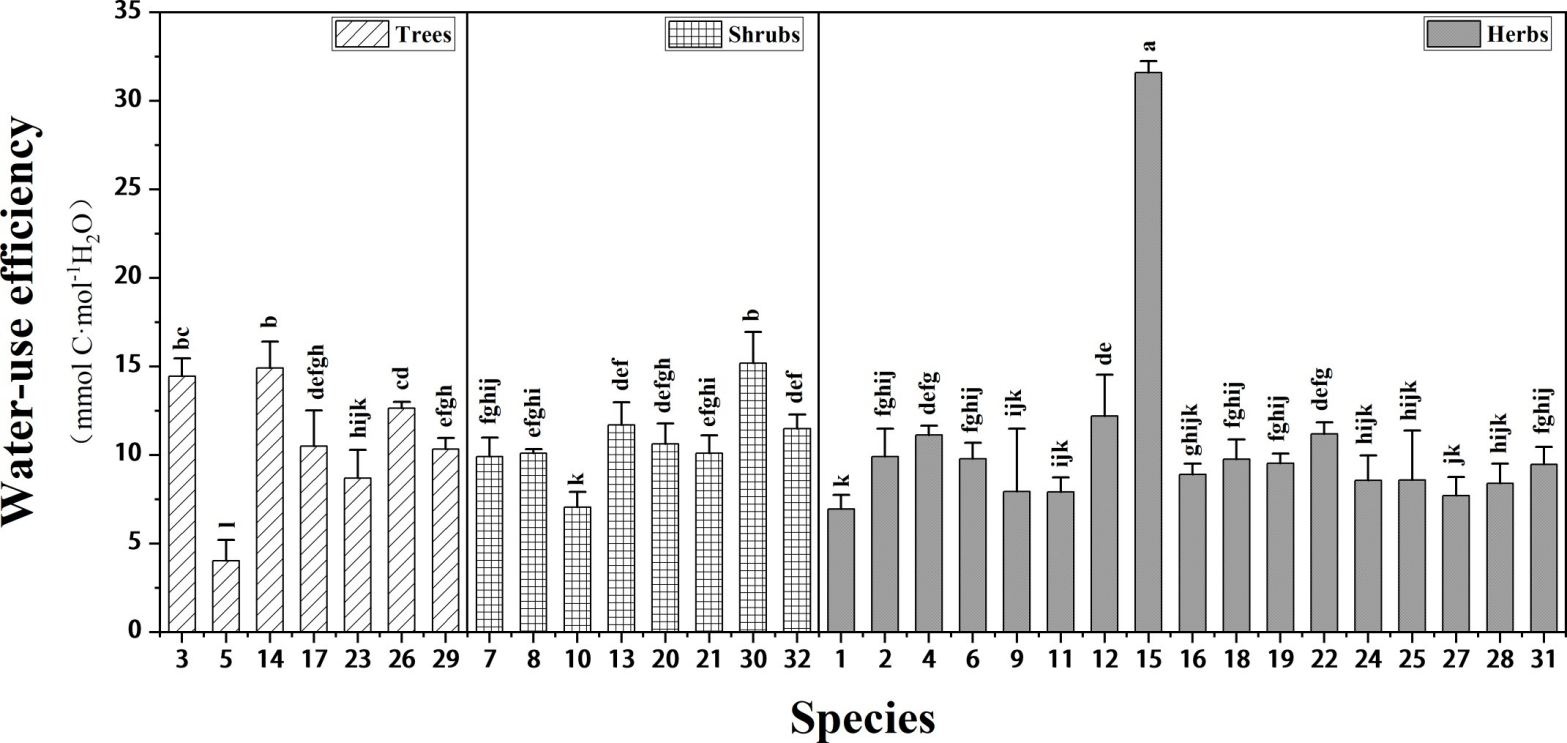
The WUE of 32 plant species surveyed. Different labels (a-l) are significantly different between the WUE of plant species (P < 0.05). The names of the species corresponding to the species numbers are shown in the notes of Fig 2.

The WUE values of 32 plant species, as shown in Fig 5, varied significantly (F = 42.313, P < 0.001), and they were between 1.6 mmol C·mol^−1^ H_2_O and 30.8 mmol C·mol^−1^ H_2_O. After multiple comparisons, it was found that the WUE of 5-*Ulmus pumila* ‘Jinye’ was significantly lower than that of all other species; while the WUE of 15-*E*. *crus-galli* was significantly higher than those of all other species.

**Fig 6:**
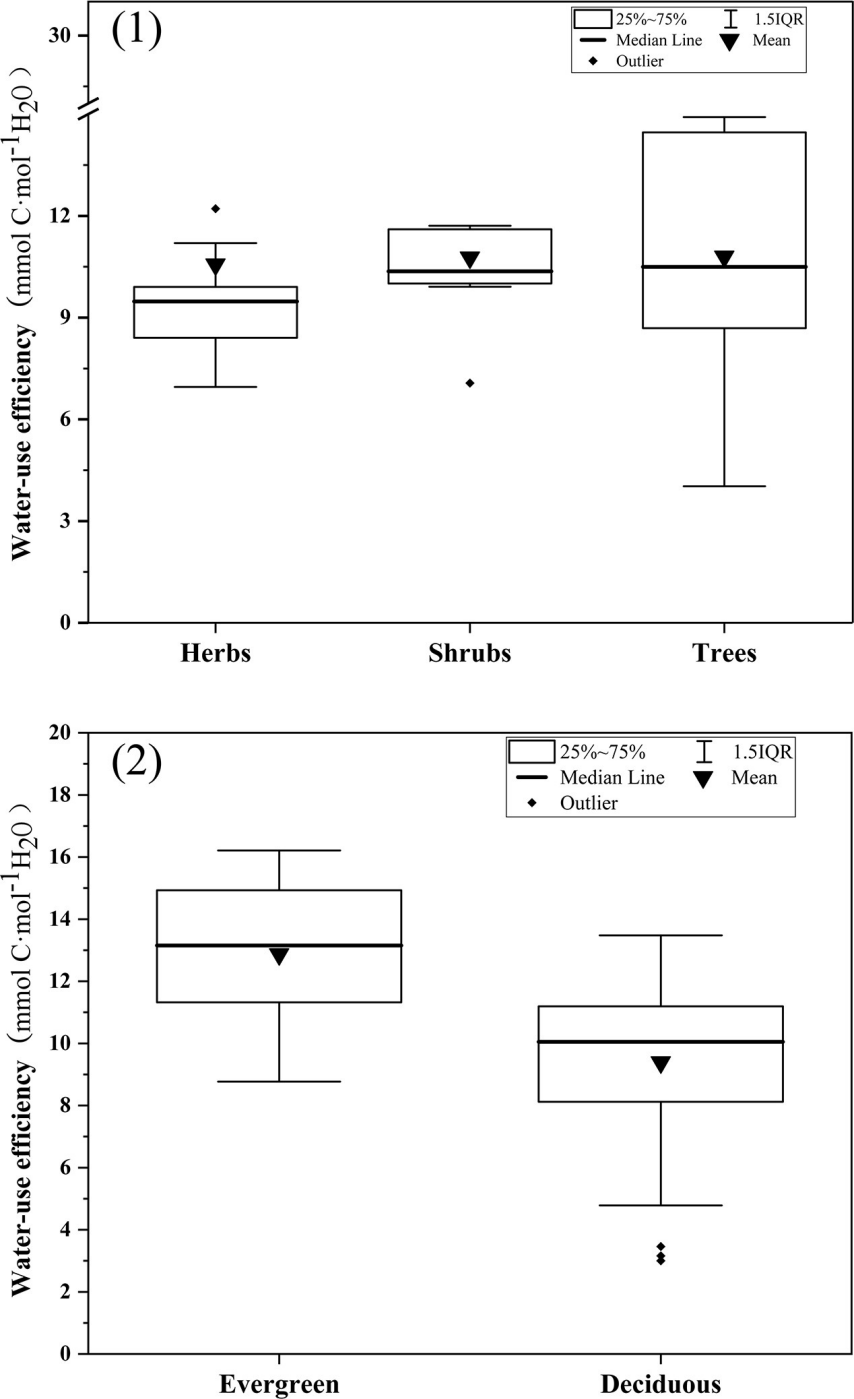
The WUE of different plant life forms. (1) the WUE of herbs, shrubs and trees; (2) the WUE of evergreen plants and deciduous plants.

The studied plants were classified as herbs, shrubs, and trees and further classified as evergreen or deciduous plants (Fig 6). There were no significant differences in the WUE of herbs, shrubs, and trees or in WUE of evergreen plants and deciduous plants. This indicates that the WUE values did not differ significantly between different plant life forms.

### Interaction effect of habitats and species identity on WUE

The two-way ANOVA results show that the species identity had a significant effect on WUE (P = 0.00), while the effect of different habitats on WUE did not differ significantly between species (P = 0.06). The interaction between habitat and species identity had a significant effect on WUE.

### Intraspecific differences in the WUE of plants

By analyzing the coefficient of variation of each species, it was found that the coefficient of variation of most species was less than 20%, and only three species had a coefficient of variation of more than 20% (Table 2); thus, the degree of variation within a species was relatively small.

**Table 2:** The mean, SD (standard deviation) and CV (coefficient of variation) of the WUE of the sampled species.

Species	Mean	SD	CV (%)	Species	Mean	SD	CV (%)
1	6.96	0.79	11.32	17	10.50	2.02	19.21
2	9.91	1.58	15.97	18	9.77	1.11	11.35
3	14.46	1.00	6.94	19	9.53	0.54	5.71
4	11.14	0.52	4.66	20	10.63	1.16	10.88
5	4.03	1.19	29.44	21	10.10	1.01	10.03
6	9.80	0.89	9.05	22	11.20	0.64	5.72
7	9.91	1.07	10.83	23	8.69	1.60	18.46
8	10.10	0.23	2.30	24	8.57	1.41	16.44
9	7.94	3.55	44.75	25	8.59	2.79	32.47
10	7.07	0.86	12.10	26	12.66	0.35	2.74
11	7.93	0.82	10.35	27	7.71	1.06	13.77
12	12.21	2.32	19.03	28	8.40	1.11	13.19
13	11.71	1.27	10.86	29	10.34	0.63	6.09
14	14.91	1.50	10.03	30	15.18	1.77	11.63
15	31.60	0.64	2.03	31	9.48	0.98	10.38
16	8.90	0.63	7.03	32	11.50	0.78	6.81

The names of the species corresponding to the species numbers are shown in the notes of Fig 2.

## Discussion

The choice of plants in sponge cities is especially important because sponge cities, like Guyuan, often face water shortages, and water availability is a major limiting factor in community structure and ecosystem function. High WUE guarantees the healthy growth of plants and the maintenance of good ecological functions. Therefore, WUE is an important index that should be considered when selecting plants for water-deficient sponge cities.

Habitat had a significant effect on the WUE of plant community; however, if the species in different habitats are the same, the effect of habitat on WUE is non-significant. This illustrates that habitat can affect WUE mainly depending on the species composition within the habitat. Therefore, we can improve the WUE of green spaces by selecting suitable plant species. Our results are consistent with those of a previous study in which the WUE in dryland habitats were optimized by selecting a specific mixture of plant species [38].

Some studies have shown that habitat or other environmental factors might have a significant effect on WUE or carbon isotope composition [39, 41, 42], mainly due to large differences in habitat geography, soil properties, or climate. For example, it was found that soil salinity and nutrient conditions had effects on plant carbon isotope composition through greenhouse control experiments or searching for habitats with significant differences [43, 44]. However, the soil properties and climate differences are usually not big among different habitats in one city; therefore, the conclusions of this study are reliable, and the WUE of green spaces can be improved by utilizing suitable plant species.

Consistent with the results of previous studies [45–47], the significant effect of species identity on WUE indicated that WUE was mainly controlled by genetic factors. The lack of significant differences among the WUE of herbs, shrubs, and trees and between evergreen and deciduous plants suggest that WUE might not differ significantly between plant life forms in a small area. The coefficient of variation of most species was less than 20%, showing that the change of sampling plots had a negligible effect on WUE for most species. The results of this study illustrate that WUE differs mainly between species, instead of different habitats or different plant life forms. Therefore, the WUE is a relatively stable indicator. To improve the WUE of green areas, the key is to select the correct species of plants.

The significant interaction between species identity and habitat indicates that the effect of habitat on WUE might depend on the species composition within the habitat and the effect of species identity on WUE might differ between habitats. The former indication corroborates our conclusion that the WUE of green spaces could be altered by utilizing different plant species, and the latter indication explains why the coefficient of variation of several species is relatively high.

WUE is a relatively stable indicator that is mainly influenced by the plant species and less by other factors; therefore, plants can be selected by comparing their WUE (choosing plants with high WUE), and in so doing, the WUE of green spaces can be improved. The average carbon isotope of plants (-28.74 ‰) [48] surveyed worldwide was converted to the WUE value, and the plants surveyed were divided into two categories according to this value: (1) higher WUE plant species and (2) the lower WUE plant species. The 1-*Verbena officinalis* L., 5-*Ulmus pumila* ‘Jinye’, 9-*Iris tectorum* Maxim., 10-*Prunus × cisterna* ‘Pissardii’, 11-*Zoysia tenuifolia*, 16-*Lavandula angustifolia* Mill., 23*-Salix matsudana* Koidz., 24-*Lythrum salicaria* L., 25-*Typha orientalis* C. Presl, 27-*Rudbeckia hirta* L., and 28-*Artemisia mongolica* (Fisch. ex Besser) Fisch. ex Nakai were classified as lower WUE plant species, and the remaining twenty-one species were classified as higher WUE plant species. The WUE of 15-*E*. *crus-galli*, in particular, was much higher than that of other species, mainly because it is a C_4_ plant [49, 50]. The WUE of C_4_ plants is generally higher than that of C_3_ plants [51, 52], and so C_4_ plants might be taken into account when selecting plants for improving the WUE of the plant community. Since Guyuan has poor soil characteristics and low rainfall, the twenty-one higher WUE plant species could be considered to select to ensure healthy plant coverage and improve the WUE of the green space.

In practical applications, when selecting greening plants for sponge cities, priority can be given to the class of higher WUE plant species that have strong adaptability to water-deficient environments and better ecological functions, while the lower WUE plant species can only be planted in relatively humid environment. Furthermore, the plant species with higher WUE can be utilized to improve the WUE of green spaces. This method is also suitable for plant selecting in non-sponge cities when the WUE is considered.

To our knowledge, this study is the first to select plant species for sponge city by using stable carbon isotope technology, providing a scientific method for rapid plant species selection for the construction of large-scale sponge cities.

## Supporting information

S1 Table The geographic coordinates of sampling plots. (DOCX)

S2 Table The names of the 32 plant species studied. (DOCX)

S3 Table The data set of plant δ^13^C and WUE. (XLSX)

S4 Table The data set of soil indexes. (XLSX)
