# Supplementary material for: Isotope-based water-use efficiency of major greening plants in a sponge city in northern China
Source: PLoS One. 2019 Jul 25;14(7):e0220083. doi: 10.1371/journal.pone.0220083 (PMC6657868; doi:10.1371/journal.pone.0220083)
Supplement: S1 Table — (DOCX) [file pone.0220083.s001.docx]

**S1 Table. The geographic coordinates of sampling plots.**

| Habitat | Geographical coordinate | Habitat | Geographical coordinate |
| --- | --- | --- | --- |
| A | 35°59.824’N, 106°12.991'E | B | 35°59.503’N, 106°13.158'E |
| A | 35°59.818’N, 106°12.976'E | B | 35°59.905’N, 106°13.397'E |
| A | 35°59.826’N, 106°12.976'E | C | 36°00.938’N, 106°16.615'E |
| A | 35°59.848’N, 106°12.920'E | C | 36°00.921’N, 106°16.595'E |
| A | 35°59.846’N, 106°12.924'E | C | 36°00.902’N, 106°16.586'E |
| A | 35°59.846’N, 106°12.911'E | C | 36°00.896’N, 106°16.583'E |
| A | 35°59.844’N, 106°13.042'E | C | 36°00.910’N, 106°16.593'E |
| A | 35°59.820’N, 106°13.072'E | C | 36°00.902’N, 106°16.589'E |
| A | 36°00.066’N, 106°13.028'E | C | 36°00.862’N, 106°16.582'E |
| A | 36°00.053’N, 106°13.043'E | C | 36°00.812’N, 106°16.559'E |
| A | 36°00.021’N, 106°13.045'E | C | 35°59.691’N, 106°13.092'E |
| A | 36°00.031’N, 106°12.996'E | C | 35°59.673’N, 106°13.071'E |
| B | 36°00.077’N, 106°13.104'E | C | 35°59.677’N, 106°13.068'E |
| B | 36°00.019’N, 106°13.109'E | C | 35°59.672’N, 106°12.990'E |
| B | 35°59.956’N, 106°13.104'E | D | 36°01.211’N, 106°17.859'E |
| B | 35°59.923’N, 106°13.110'E | D | 36°01.130’N, 106°17.888'E |
| B | 35°59.509’N, 106°13.141'E | D | 36°01.081’N, 106°17.910'E |
| B | 35°59.505’N, 106°13.167'E | D | 36°01.012’N, 106°17.907'E |
| B | 35°59.501’N, 106°13.182'E |  |  |

A, B, C and D represent residential green spaces, street green spaces, park green spaces and the riverside wetland respectively.
