# Supplementary material for: Isotope-based water-use efficiency of major greening plants in a sponge city in northern China
Source: PLoS One. 2019 Jul 25;14(7):e0220083. doi: 10.1371/journal.pone.0220083 (PMC6657868; doi:10.1371/journal.pone.0220083)
Supplement: S2 Table — (DOCX) [file pone.0220083.s002.docx]

**S2 Table. The names of the 32 plant species studied.**

| Species number | Plant species name |
| --- | --- |
| 1 | *Verbena officinalis* L. |
| 2 | *Cirsium* *setosum* (Willd.) Besser ex M.Bieb. |
| 3 | *Picea asperata* Mast. |
| 4 | [*Dianthus* *chinensis* L.](http://www.theplantlist.org/tpl1.1/record/kew-2764027) |
| 5 | *Ulmus pumila* ‘Jinye’ |
| 6 | *Rumex acetosa* L. |
| 7 | *Rosa chinensis* Jacq. var. *spontanea* (Rehd. et Wils.) Yü et Ku |
| 8 | *Tamarix ramosissima* Lbd*.* |
| 9 | *Iris tectorum* Maxim. |
| 10 | *Prunus × cisterna* ‘Pissardii’ |
| 11 | *Zoysia tenuifolia* |
| 12 | *Sedum spectabile* |
| 13 | *Amygdalus triloba* (Lindl.) Ricker |
| 14 | *Juniperus* *formosana* Hayata |
| 15 | *E. crus-galli* |
| 16 | *Lavandula angustifolia* Mill. |
| 17 | *Prunus Cerasifera Ehrh. f. atropurpurea* (Jacq.) Rehd. |
| 18 | *Trifolium repens* L. |
| 19 | *Iris lacteal Pall. var. chinensis* (Fisch.) Koidz. |
| 20 | *Rosa xanthina* Lindl. |
| 21 | *Euonymus phellomanus* Loes. |
| 22 | *Hosta ventricosa* (Salisb.) Stearn |
| 23 | *Salix matsudana* Koidz. |
| 24 | *Lythrum salicaria* L. |
| 25 | [*Typha* *orientalis* C. Presl](http://www.theplantlist.org/tpl1.1/record/kew-271046) |
| 26 | [*Platycladus* *orientalis* (L.) Franco](http://www.theplantlist.org/tpl1.1/record/kew-2411523) |
| 27 | *Rudbeckia hirta* L. |
| 28 | [*Artemisia* *mongolica* (Fisch. ex Besser) Fisch. ex Nakai](http://www.theplantlist.org/tpl1.1/record/gcc-88993) |
| 29 | [*Salix* *babylonica* L.](http://www.theplantlist.org/tpl1.1/record/kew-5004552) |
| 30 | [*Forsythia* *viridissima* Lindl.](http://www.theplantlist.org/tpl1.1/record/kew-369455) |
| 31 | *Coreopsis lanceolata* L. |
| 32 | [*Syringa* *oblata* Lindl.](http://www.theplantlist.org/tpl1.1/record/kew-356313) |
